# Towards evidence-based vitamin D supplementation in infants: vitamin D intervention in infants (VIDI) — study design and methods of a randomised controlled double-blinded intervention study

**DOI:** 10.1186/s12887-017-0845-5

**Published:** 2017-03-29

**Authors:** Otto Helve, Heli Viljakainen, Elisa Holmlund-Suila, Jenni Rosendahl, Helena Hauta-alus, Maria Enlund-Cerullo, Saara Valkama, Kati Heinonen, Katri Räikkönen, Timo Hytinantti, Outi Mäkitie, Sture Andersson

**Affiliations:** 1 Children’s Hospital, Pediatric Research Center, University of Helsinki and Helsinki University Hospital, P.O. Box 281, 00029 HUS Helsinki, Finland; 2 Folkhälsan Research Center, Helsinki, Finland; 3 Institute of Behavioral Sciences, University of Helsinki, Helsinki, Finland; 4 Center for Molecular Medicine, Karolinska Institutet and Clinical Genetics, Karolinska University Hospital, Stockholm, Sweden

**Keywords:** Allergy, Asthma, Bone development, Cognitive development, Immunity, Infants, Infections, Vitamin D

## Abstract

**Background:**

Vitamin D is important for bone mass accrual during growth. Additionally, it is considered a requirement for a multitude of processes associated with, for example, the development of immunity. Many countries apply vitamin D supplementation strategies in infants, but the guidelines are not based on scientific evidence and aim at prevention of rickets. It remains unclear whether the recommended doses are sufficient for the wide array of other effects of vitamin D. The VIDI trial performed in Finland is the first large randomised controlled study for evaluation of the effects of different vitamin D supplemental doses in infancy on:

1. bone strength
2. infections and immunity
3. allergy, atopy and asthma
4. cognitive development
5. genetic regulation of mineral homeostasis

**Methods/Design:**

VIDI, a randomised controlled double-blinded single-centre intervention study is conducted in infants from the age of 2 weeks to 24 months. Participants, recruited at Helsinki Maternity Hospital, are randomised to receive daily either 10 μg (400 IU) or 30 μg (1 200 IU) of vitamin D3 supplementation. Both groups are assessed at 6 months of age for calcium homeostasis, and at 12 and 24 months of age for parameters associated with bone strength, growth, developmental milestones, infections, immunity, atopy-related diseases, and genetic factors involved in these functions.

**Discussion:**

The study enables evaluation of short and long term effects of supplemental vitamin D on growth, immune functions and skeletal and developmental parameters in infants, and the effects of genetic factors therein. The results enable institution of evidence-based guidelines for vitamin D supplementation in infancy.

**Trial registration:**

ClinicalTrials.gov, NCT01723852, registration date 6.11.2012.

**Electronic supplementary material:**

The online version of this article (doi:10.1186/s12887-017-0845-5) contains supplementary material, which is available to authorized users.

## Background

The biologically inactive vitamin D_3_ (cholecalciferol) is produced in the skin after solar UVB exposure. It is bound to vitamin D binding protein (DBP) and transported to the liver for conversion to 25-hydroxy-vitamin D (25-OHD), the most abundant circulating metabolite of vitamin D. In the kidney, 25-OHD is further hydroxylated to the active form calcitriol, 1,25-OH_2_D, that contributes to calcium metabolism [1]. Paracrine conversion of 25-OHD to calcitriol occurs in many tissues, such as dendritic and endothelial cells, brain, placenta and parathyroids, underscoring the importance of vitamin D for these organs [2]. Based on the number of cell types expressing vitamin D receptors (VDR), 38 potential target tissues have been recognised [3]. In target cells, calcitriol regulates the transcription of target genes, including several genes involved in cell cycle regulation [4]. Locally produced calcitriol in immune cells participates in host defence reactions and acts as a potent immunomodulator [2, 5]. In fact, vitamin D has been estimated to directly and indirectly regulate the expression of 2–3% of the human genome [4].

Both cutaneously synthesised and dietary vitamin D contribute to the circulating 25-OHD concentration. There is no consensus on the optimal 25-OHD concentration. In 2011, Institute of Medicine guideline stated that 25-OHD concentrations above 50 nmol/L are required for normal body functions including linear growth and bone mass accrual [6]. According to the Endocrine Society [7], for optimizing long-term health benefits such as the prevention of diabetes or fractures, concentrations above 75 nmol/L may be needed.

Vitamin D supplementation has been recommended in Finland for all infants since the 1940’s, but in line with the declining prevalence of rickets, the recommended doses have gradually decreased. The present Finnish Nutritional Council guidelines recommend 10 μg (400 IU) of vitamin D3 supplementation daily for all infants from the age of 2 weeks to 24 months, and 7.5 μg (300 IU) to children aged 2 to 18 years. Despite these recommendations, 20% of children were shown to be vitamin D deficient (<50 nmol/L) at 14 months of age [8]. In addition, we have previously shown that more than 70% of 195 apparently healthy school children in Helsinki, Finland, were vitamin D deficient (<50 nmol/L) during the school year [9, 10].

### Role of vitamin D in health and disease

#### Growth and bone mineralisation

Serum calcium and phosphate concentrations are tightly regulated by parathyroid hormone (PTH) and 1,25-OH_2_D by negative feedback. Rickets is caused by insufficient vitamin D supply resulting in the lack of sufficient calcium and phosphate for bone mineralization at the growth plates [11]. The worldwide incidence of rickets is increasing [11]. Vitamin D deficiency is associated with low bone mineral density (BMD) and an unfavourable bone development in children aged 6–16 years and in newborns [12, 13]. However, data on the relationship between vitamin D and BMD in children are somewhat contradictory. In healthy children aged 8–17 years with S-25-OHD levels above 50 nmol/L, no effect of vitamin D supplementation on BMD is seen [14], and the relationship between BMD and S-25-OHD is inconsistent in infants and young children [15].

In infants, severe vitamin D deficiency (<25 nmol/L) associates with poor linear growth and delayed motor development possibly due to muscle weakness [16, 17]. In vitamin D deficient children, vitamin D supplementation improved growth velocity and prevented stunted growth [18, 19]. The increase in S-25-OHD concentration increased circulating IGF-1 level [18].

#### Infections and immunity

Rickets commonly presents with comorbidities such as continuous or recurrent respiratory infections [20, 21]. Vitamin D has profound effects on the immune system by promoting immune responses and inducing innate immune defences. Immune cells express vitamin D receptor (VDR) and respond to vitamin D [22, 23]. On innate immunity, vitamin D inhibits the expression of pattern-recognition receptors, induces autophagy in macrophages and induces endogenous antimicrobial peptides, such as cathelicidin. On adaptive immunity, for example, vitamin D acts by regulating the release of pro-inflammatory cytokines from mononuclear cells and inhibits T-cell proliferation through decreased Th1 cytokine secretion [24–26]. Therefore, vitamin D does not only have a role in modulating host defence to infections, but is likely to control factors in autoimmunity, also in allergic disease.

Vitamin D modifies response to both bacterial and viral infections [24, 27]. In adults, vitamin D insufficiency increases the risk of severe infections [28], but the results from intervention trials are conflicting [29]. Vitamin D deficiency may contribute to increased occurrence of lower respiratory tract infections in children [30]. In 167 Japanese children aged 6–16 years a supplemental vitamin D3 of 1 200 IU for a period of 4 months resulted in a significant reduction in influenza A infections [31]. In addition, vitamin D deficiency is associated with upper respiratory tract, gastrointestinal and ear infections in children [32, 33]. Identification, treatment, and prevention of vitamin D deficiency in early childhood may therefore have widespread health effects throughout childhood.

#### Allergy, atopy and asthma

In Finland, the prevalence of asthma among children aged 7–16 years is 7% [34]. Based on epidemiological studies, low maternal intake of vitamin D at pregnancy and low cord blood 25-OHD concentration have been associated with a higher risk for asthma, allergic rhinitis and atopic eczema in the offspring, as assessed at 5 years of age [35–38]. However, findings have not been consistent [39] and recent studies on prenatal vitamin D supplementation have not demonstrated an effect on wheezing at 3 years of age [40, 41]. Vitamin D may, however, have a protective effect against childhood wheezing and asthma, but the evidence is inconclusive [42]. In adults, IgE concentrations are higher for those adults with low or very high concentration of serum 25-OHD (S-25-OHD) [43].

#### Cognitive development

The association between concentrations of S-25-OHD and cognitive abilities has been widely studied among middle-aged and older individuals [44–46]. Vitamin D trials are rare and they have not been able to support beneficial effects of vitamin D supplementation on cognitive functions among adults [47, 48]. Little evidence exists on the effects of vitamin D on the cognitive development among children and adolescents. Low concentrations of cord blood 25-OHD increased the risk for delayed mental and psychomotor development in 363 16–18-months-old children with severe vitamin D deficiency [49]. Surprisingly, also the children with the highest S-25-OHD concentrations had deficits in psychomotor development. However, S-25-OHD concentrations were not associated with cognitive performance in adolescents after controlling for ethnicity [50–52]. Although preliminary results point to importance of early life exposure, the role of vitamin D in cognitive development during childhood and adolescence remains largely unstudied.

#### Genetic regulation of mineral homeostasis

S-25-OHD concentration has high heritability (28–80%), which explains differences in responses to vitamin D supplementation [53]. Genome-wide association studies on vitamin D insufficiency identified a number of common genetic variants that influence 25-OHD concentrations and the risk of insufficiency [54]. These included single nucleotide polymorphisms in the GC gene encoding vitamin D binding protein, locus near the 7-dehydrocholesterol reductase involved in vitamin D synthesis in the skin, and CYP2R1 coding cytochrome P450 responsible for 25-hydroxylation. We have studied GC polymorphisms in relation to vitamin D metabolism and bone strength in children [55] and observed differences in S-25-OHD and PTH concentrations between GC genotypes and that variation exists in BMD and bone strength index as well. The variation in S-25-OHD between GC genotypes suggests differences in vitamin D utilisation. Polymorphisms of the vitamin D receptor gene VDR associate with growth pattern in infancy [56, 57] and with bone strength [58]. VDR polymorphisms together with vitamin D supplementation affect bone mineral accrual suggesting that vitamin D supplementation could be used to change the developmental trajectory and induce phenotype with lowered risk for chronic disease or disorder [59, 60]. Other possible candidate genes include CYP24A1 that encodes 24-hydroxylase initiating degradation of both 25-OHD and 1,25-OHD [54].

### Overall aims and research questions

The aim of the study is to investigate long-term health benefits of early vitamin D supplementation with a dose ensuring effective concentration of 25-OHD.

The primary outcomes of the study are bone strength and cumulative frequency of infections at 24 months of age. We hypothesise that a higher vitamin D supplementation to infants between 2 weeks and 24 months of age will increase bone strength and decrease the frequency of infections.

Secondary outcomes of the study are occurrence of IgE mediated allergic symptoms, growth, motor, cognitive and social development, and associations of concentration and effects of vitamin D with polymorphisms of genes associated with its synthesis, binding proteins and degradation.

## Methods/Design

### Study design and setting

**Fig. 1:**
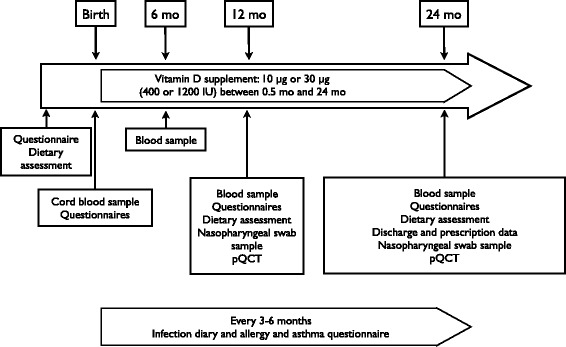
Study protocol. Methods are listed in Table 1 and further described in Additional documentation

This is a randomised on-going controlled double-blinded intervention trial on children of 2 weeks to 24 months of age (Fig. 1). Vitamin D supplementation was commenced at 2 weeks of age and continued until 24 months of age.

This study is carried out at the Kätilöopisto Maternity Hospital, Helsinki, Finland (60°20,40’N). The hospital is one of three hospitals providing maternity services for the greater Helsinki area (a population of 1.5 million) with 8 000 births annually (15 000 in the whole greater Helsinki area).

### Research team

The research team responsible for inception, implementation, and management of the protocol includes paediatricians, paediatric specialists in the field of neonatology, endocrinology, infectious diseases and immunology, psychologists, and nutritionists. The research team and the research nurses undertake recruitment, data collection, follow-up visits, and data entry.

### Procedure and participants

A total of 4 980 infants were assessed for eligibility and 1 572 did not meet the inclusion criteria. Written information of the study was given to 3 408 families at the Helsinki Maternity Hospital 1–2 days after delivery from which 2 421 declined to participate. Altogether 987 healthy term newborns were recruited between January 2013 and June 2014.

The participating mothers were of Caucasian origin, without regular medication and with a singleton pregnancy. The infants were born at term (37 + 0 to 42 + 0 weeks) with a birth weight appropriate for gestational age (birth weight SD score between −2.0 to +2.0). Exclusion criteria for the infants were nasal continuous positive airway pressure treatment for more than 1 day, intravenous glucose infusion, intravenous postnatal antibiotic treatment, seizures, duration of phototherapy for more than 3 days, and need for nasogastric tube for more than 1 day.

Girls (492) and boys (495) were randomised at recruitment in blocks of 50 to receive either 10 or 30 μg of vitamin D3 (400 IU or 1 200 IU, respectively) daily during the study period. Randomisation was performed by an external pharmacist and was blinded from the study group.

Vitamin D is administered once daily with 5 drops for both concentrations. The families received a study diary to record treatment compliance prospectively and empty vitamin D bottles are collected at follow-up visits.

Each family keep record of the child’s infections on a study diary throughout the study period. Data on allergic symptoms, growth, child’s motor and neurocognitive functioning, mental and behavioural health, social skills, sleep and diet are also collected. Furthermore, mothers report their own well-being at child’s birth (questionnaires given at recruitment) and at 12- and 24-month follow-up. Follow-up visits for the children are arranged at 6, 12 and 24 months of age at which blood samples are taken. Bone strength is measured with peripheral quantitative computer tomography (pQCT) at 12 and 24-month follow-up visits (overview of study follow-up and parameters in Table 1). During the visit the families meet a study nurse and have the possibility to meet with a paediatrician.

**Table 1:** Methods and timing of sampling. Methods are characterised further in Additional documentation

	Birth	6 months of age	12 months of age	24 months of age
Vitamin D	x		x	x
Calcium and bone metabolism	x	x	x	x
Inflammatory markers	x		x	x
DNA	x		x	x
Bone densitometry			x	x
Infection records		x	x	x
Allergy questionnaire		x	x	x
Specific allergic responses (IgA, IgE)			x	x
Iron status			x	x
Dietary survey	x^a^	x	x	x
Neurological scales			x	x
Family sociodemographic and lifestyle	x			x
Neurocognitive development scales			x	x
Mental health and behaviour	x		x	x
Clinical assessment		x	x	x
Health records	x	x	x	x
Viral and bacterial swab			x	x

^a^retrospectively on maternal diet

**Fig. 2:**
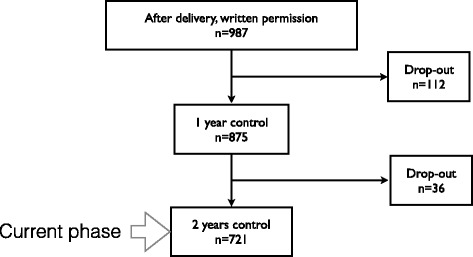
Study recruitment, adherence, and current phase of the study

A total of 875 participants have been to their 12-month follow-up visit (a drop-out of 112 children) and the 24-month follow-up visits are ongoing (to date, parents of 36 children have declined to participate in the 24-month follow-up visit, Fig. 2).

### Ethical considerations

An informed consent was obtained from the parents at recruitment. Previous intervention studies have evaluated the safety of substitution doses of up to 50 μg vitamin D in infants [61]. Safety was ensured by performing a pilot study for the proposed project: 113 children were randomised into three groups receiving vitamin D3 doses of 10, 30 and 40 μg daily for 3 months [62]. No hypercalcemia was observed in this study. Plasma and urine calcium concentrations did not differ between the three groups and in all groups the mean concentration of S-25-OHD reached at least 80 nmol/L, which was considered sufficient. Therefore, 10 μg a day was considered adequate and ethically sound to use as the dosage for the control group. While the group receiving 40 μg/day did not develop hypercalcaemia, their S-25-OHD reached levels higher than targeted. Dose of 30 μg/day was therefore chosen for the present study.

An external steering group was recruited to monitor the study and possible adverse effects.

The vitamin D3 supplements are prepared by Orion Pharmaceuticals (Espoo, Finland). The study is researcher initiated and independent.

The Research Ethics Committee of the Hospital District of Helsinki and Uusimaa has approved the study (ID 107/13/03/03/2012) and it is registered into ClinicalTrials.com (NCT01723852).

### Study measures

An overview of study parameters is shown in Table 1. A full description of specific methods used in the study are presented in the online Additional documentation. In brief, the parameters used for the assessment of primary outcomes are as follows: bone parameters assessed with pQCT at 12 and 24 months of age, daily infection diary filled in by parents from 2 weeks to 24 months of age, and S-25-OHD at birth (cord blood) and at 12 and 24 months of age.

### Data analyses

Differences between groups are tested with relevant statistical models, e.g., independent samples *t*-test, ANOVA and analysis of covariance (ANCOVA). Temporal change and difference between intervention groups of continuous variables (i.e., S-25-OHD) are assessed with repeated measures ANCOVA. Furthermore, multivariate and multilevel regression models adjusted with potential confounders are applied to explore associations between several outcomes when applicable.

### Sample size

On the basis of sample size calculation for both of the primary outcomes, 400 + 400 study subjects, allowing for a 20% drop-out rate, a 0.2 SD difference can be detected with p at 0.05 and ß of 0.9. In bone mineral content, a 0.2 SD increase requires 210 + 210 subjects, and a similar increase in bone cross sectional area 297 + 297 subjects as measured by the same technique as here (pQCT) [63]. The annual rate of infections in children below 2 years of age is 6 (SD ± 1.6) [64, 65]. In the group receiving 30 μg of vitamin D we expect a decrease from 12 to 9 infections during the 24-month study period. A sample size of 220 + 220 is required to achieve statistical power.

## Discussion

To the best of our knowledge, this is the largest and most comprehensive randomised intervention study to assess the effects of vitamin D supplementation on skeletal and extra skeletal outcomes in infants. To date, epidemiological studies and randomised intervention trials have demonstrated conflicting results on, for example, the effect of vitamin D supplementation on childhood infections and on neurocognitive functioning. This may be due to difficulties in addressing the multiple confounding factors affecting the association between exposure and outcome. The present study, based on findings from a pilot study [62], is designated to specific hypothesis with adequate statistical power and duration. During the study, we gather data on infant diet at home and day care, psychological profiles and atopy-related symptoms using questionnaires. Results from this study will likely generate novel evidence-based information for improving vitamin D status among the paediatric population worldwide. By analysis of the impact of gene polymorphisms on the concentration and efficacy of the given vitamin D substitution it may create the basis for individualised dosage guidelines.

### Limitations of the study

Compliance of the study subjects is always an issue in intervention trials. We are collecting used vitamin D bottles to evaluate the amount of vitamin D left after each cycle. These data, and parentally kept diaries on the use of vitamin D are used to evaluate the compliance in vitamin D intake.

Questionnaires may be improperly filled, resulting in missing data on, for example, parent-reported infections. Therefore, national registers can be used for the validation of caregiver data.

The results from a study performed in Finland cannot be directly generalised to other parts of the world. However, due to the randomised controlled study design our study will provide valuable data on the effects of vitamin D substitution in populations with variable vitamin D status. In addition, the study is designed to have control over the most common sources of vitamin D.

If the results from the VIDI-trial confirm the connections between the increased dose of vitamin D and the primary outcomes, the health benefits gained by a population-wide supplementation with higher vitamin D dose — a simple and low-cost intervention — are obvious. The first results of the VIDI-trial are expected in 2017.

## Additional file

Additional file 1: Supplementary documentation. Methods described in further detail. (DOCX 520 kb)

## Abbreviations

25-OHD: 25-hydroxy-vitamin D
BMD: Bone mineral density
DBP: Vitamin D binding protein
S-25-OHD: serum 25-OHD
VDR: Vitamin D receptor
