# Supplementary material for: Towards evidence-based vitamin D supplementation in infants: vitamin D intervention in infants (VIDI) — study design and methods of a randomised controlled double-blinded intervention study
Source: BMC Pediatr. 2017 Mar 29;17:91. doi: 10.1186/s12887-017-0845-5 (PMC5372327; doi:10.1186/s12887-017-0845-5)
Supplement: Additional file 1: — Supplementary documentation. Methods described in further detail. (DOCX 520 kb) [file 12887_2017_845_MOESM1_ESM.docx]

Additional file 1

Additional documentation, list of methods.

1. S-25OHD concentration is measured with an automated IDS-iSYS analyser (IDS Ltd., Boldon, UK). The method shows good linear agreement with liquid chromatography in tandem with mass spectrometry (LC-MS, R^2^=0.942, in-house comparison performed with 67 samples). The mean (95% CI) value for the ratio of IDS-iSYS 25-OHD to LC-MS 25-OHD concentration is 0.73 (0.68; 0.78). Intra- and inter-assay CV% for 25-OHD were < 5 % and < 8 %, respectively. Our laboratory participates in inter laboratory quality assessment scheme for vitamin D, DEQAS (Charing Cross Hospital, London UK).
2. Serum intact parathyroid hormone (S-iPTH) is measured with an automated IDS-iSYS analyser with CLIA method. The specificity for other fragments is less than 4% with intra- and interassay CVs of < 5% (own laboratory). The measured bone formation markers include e.g. bone-specific alkaline phophatase (BAP) and intact N-terminal propeptides of type I collagen (PINP), and bone resorption markers include C-terminal cross-linked telopeptides of type I collagen (CTX-I). These are measured with automated methods using the IDS-iSYS automated analyzer (IDS Ltd, Boldon, UK). Serum intact FGF23 is assayed with Kainos ELISA kit by Kainos Laboratories (Tokyo, Japan) and c-terminal fragments of FGF23 with Biomedica immunoassay (BIOMEDICA Medizinprodukte GmbH & CO KG, Vienna, Austria).
3. A bone mineral density measurement is performed to all participants with peripheral quantitative computed tomography (pQCT) from distal tibia with a XCT-2000 scanner (Stratec Medizintechnik GmbH, Pforzheim, Germany) at annual visits. pQCT gives information about volumetric bone density and geometry in the total, trabecular and cortical bone compartments. In addition, it allows for the calculation of bone strength parameters e.g. cross-sectional moments of inertia. The tibia is structurally at its weakest at 15-20 % length from the distal end [1, 2]. The physician in charge of the follow-up visit marks and measures the length of the left tibia from the medial malleolus to the medial knee joint cleft. A transverse line is drawn at 20 % of this length from the distal tibia to indicate the site for the pQCT scan. The leg being scanned is supported by a cast closed with three Velcro tapes. All analyses are performed with the integrated XCT software (version 6.00). Scan speeds are set at 22mm/s and a voxel site of 0.2 mm is used. The quality of the scans are graded visually from 1 to 5 as described elsewhere


   [3] and by us [4].
4. The studied inflammation markers include e.g. white cell count, IL-6, hs-CRP, totIgE and antigen-specific IgE. An ELISA method is used for the assessing of hs-CRP from serum samples with inter-CV 2 %: IL-6 is a cytokine increasing hs-CRP production in the liver and one of the targets of VDR. IL-6 could mediate anti-inflammatory effects of vitamin D. IL-6 is detected with flow cytometry using Luminex technique. Cathelicidin is assessed using an ELISA method. Total IgE and airway and food allergen specific IgE will be analysed according by a validated commercial laboratory (HUSLAB, Helsinki University Central Hospital, Finland).
5. At 12 and 24 monthsof age, a nasopharyngeal swab sample is obtained during the follow-up visit. Viral and bacterial pathogens and colonisation will be assessed by RT-PCR.
6. Based on previous genome-wide association studies, a number of common genetic variants relevant to vitamin D sufficiency


   [5], allergic sensitisation


   [6] and trabecular and cortical volumetric BMD


   [7] have been identified. TaqMan® SNP Genotyping Assays are utilised for genotyping. The traits are polygenic, but considering physiological status and linkage disequilibrium, we will design optimal assays and possibly combine these into diplotypes. In addition, epigenetic effects will be assessed. These analyses will be carried out in Folkhälsan Research Centre, Biomedicum, Helsinki. The population of 1000 subjects is large enough to identify clinically significant genetic variants that modify the response to the intervention.
7. Parents fill in a calendar each day where they mark each supplementation taken first with a 3-month and, after 12 months of age, 6-month interval. Data on breastfeeding, introduction of complimentary foods, use of other dietary supplements and traveling are recorded. In addition, any infection is marked and described for each day. In regard to this, visits to a physician, medications taken and possible hospital care are also described.
8. Allergy, atopy and asthma symptom questionnaires will be filled both on the Internet every 3 to 6 months (<https://elomake.helsinki.fi/lomakkeet/37011/lomake.html>, in Finnish) and at each follow-up visit.
9. Infant dietary assessment includes a three-day food record at 12 months of age and a food frequency questionnaire (FFQ) at 24 months of age. Nutrient intake at 12 months of age is calculated with AivoDiet software (Aivo Finland, Turku, Finland). In addition, the infant FFQ contains specific questions on dairy products and dietary fats for a more specific qualification. During study visits families are instructed to keep food record for three consecutive days on infant’s eating. An example of recording will be provided. Dietary pattern based on food choices are created to get an overall picture on eating behaviour.
10. At recruitment, maternal diet is evaluated with a FFQ


    [4]. The maternal retrospective FFQ contains 22 quantified food group items and the frequency of these consumed during the last month of pregnancy is assessed. The use of maternal dietary supplements is asked retrospectively from the previous 6 months of pregnancy.
11. Ages and Stages Questionnaire (ASQ) is a validated questionnaire for evaluation of motor and cognitive functions, and personal and social skills at 12 and 24 month visits. ASQ is delivered to families before study visit and families return it filled to the study nurse at their study visit. To evaluate the motor development the paediatrician utilises Griffith’s and Bayley scale.
12. Child’s mental health and behaviour is evaluated with several maternal reported questionnaires at birth, and/or at 12- and 24-month follow-ups including Neonatal Perception Inventory (NPI) a questionnaire to assess maternal perceptions regulatory behaviour of her newborn child, Infant/child Behaviour Questionnaire (IBQ/CBQ) for evaluation of child’s temperament, Infant Toddler Socio Emotional Assessment (ITSEA) for evaluation of child’s social or emotional problems and competencies, Sensory Experiences Questionnaire (SEQ) to assess child’s sensory abnormalities.
13. Maternal mental health and behaviour is evaluated with self-reported questionnaires including The Center for Epidemiologic Studies Depression Scale (CESD), the Adult Self-Report (ASR) of psychiatric symptoms and Parenting Stress Index (PSI) at birth and at 12 and 24 month follow-up.
14. Discharge and prescription data are gathered at the end of the study from national registries. The use of antimicrobials and infection related diagnoses are added to questionnaire data.

References

[1] Binkley TL, Specker BL, Wittig TA. Centile curves for bone densitometry measurements in healthy males and females ages 5-22 yr. J Clin Densitom. 2002;5:343-53.

[2] Specker B, Binkley T. Randomized trial of physical activity and calcium supplementation on bone mineral content in 3- to 5-year-old children. J Bone Miner Res. 2003;18:885-92.

[3] Blew RM, Lee VR, Farr JN, Schiferl DJ, Going SB. Standardizing evaluation of pQCT image quality in the presence of subject movement: qualitative versus quantitative assessment. Calcif Tissue Int. 2014;94:202-11.

[4] Viljakainen HT, Valta H, Lipsanen-Nyman M, Saukkonen T, Kajantie E, Andersson S, et al. Bone characteristics and their determinants in adolescents and young adults with early-onset severe obesity. Calcif Tissue Int. 2015;97:364-75.

[5] Wang TJ, Zhang F, Richards JB, Kestenbaum B, van Meurs JB, Berry D, et al. Common genetic determinants of vitamin D insufficiency: a genome-wide association study. Lancet. 2010;376:180-8.

[6] Bonnelykke K, Matheson MC, Pers TH, Granell R, Strachan DP, Alves AC, et al. Meta-analysis of genome-wide association studies identifies ten loci influencing allergic sensitization. Nat Genet. 2013;45:902-6.

[7] Paternoster L, Lorentzon M, Lehtimaki T, Eriksson J, Kahonen M, Raitakari O, et al. Genetic determinants of trabecular and cortical volumetric bone mineral densities and bone microstructure. PLoS Genet. 2013;9:e1003247.
